# The Effects of Environmental Exposure on Epigenetic Modifications in Allergic Diseases

**DOI:** 10.3390/medicina60010110

**Published:** 2024-01-07

**Authors:** Sandra Mijač, Ivana Banić, Ana-Marija Genc, Marcel Lipej, Mirjana Turkalj

**Affiliations:** 1 Department of Medical Research, Srebrnjak Children’s Hospital, Srebrnjak 100, HR-10000 Zagreb, Croatia; smijac@bolnica-srebrnjak.hr (S.M.); amgenc@bolnica-srebrnjak.hr (A.-M.G.); 2 Department of Innovative Diagnostics, Srebrnjak Children’s Hospital, Srebrnjak 100, HR-10000 Zagreb, Croatia; 3 IT Department, Srebrnjak Children’s Hospital, Srebrnjak 100, HR-10000 Zagreb, Croatia; mlipej@bolnica-srebrnjak.hr; 4 Department of Pediatric Allergy and Pulmonology, Srebrnjak Children’s Hospital, Srebrnjak 100, HR-10000 Zagreb, Croatia; mturkalj@bolnica-srebrnjak.hr; 5 Faculty of Medicine, J.J. Strossmayer University of Osijek, J. Huttlera 4, HR-31000 Osijek, Croatia; 6 Faculty of Medicine, Catholic University of Croatia, Ilica 242, HR-10000 Zagreb, Croatia

**Keywords:** allergy, epigenetic modifications, environmental exposure, immune regulation, DNA methylation, miRNA expression, asthma, histone modifications

## Abstract

Allergic diseases are one of the most common chronic conditions and their prevalence is on the rise. Environmental exposure, primarily prenatal and early life influences, affect the risk for the development and specific phenotypes of allergic diseases via epigenetic mechanisms. Exposure to pollutants, microorganisms and parasites, tobacco smoke and certain aspects of diet are known to drive epigenetic changes that are essential for immune regulation (e.g., the shift toward T helper 2-Th2 cell polarization and decrease in regulatory T-cell (Treg) differentiation). DNA methylation and histone modifications can modify immune programming related to either pro-allergic interleukin 4 (IL-4), interleukin 13 (IL-13) or counter-regulatory interferon γ (IFN-γ) production. Differential expression of small non-coding RNAs has also been linked to the risk for allergic diseases and associated with air pollution. Certain exposures and associated epigenetic mechanisms play a role in the susceptibility to allergic conditions and specific clinical manifestations of the disease, while others are thought to have a protective role against the development of allergic diseases, such as maternal and early postnatal microbial diversity, maternal helminth infections and dietary supplementation with polyunsaturated fatty acids and vitamin D. Epigenetic mechanisms are also known to be involved in mediating the response to common treatment in allergic diseases, for example, changes in histone acetylation of proinflammatory genes and in the expression of certain microRNAs are associated with the response to inhaled corticosteroids in asthma. Gaining better insight into the epigenetic regulation of allergic diseases may ultimately lead to significant improvements in the management of these conditions, earlier and more precise diagnostics, optimization of current treatment regimes, and the implementation of novel therapeutic options and prevention strategies in the near future.

## 1. Introduction

Epigenetics is defined as the study of the interaction of the environment and the functioning of the genome without changing the genome sequence. Epigenetic modifications are (usually reversible) changes of the genome (chromatin) and are functionally relevant, but do not affect the nucleotide sequence of the DNA. These include DNA methylation and histone modifications. Additionally, certain post-transcriptional control elements, such as the expression of non-coding RNAs, are also considered important epigenetic regulators.

DNA methylation implies the addition of the methyl group to the cytosine nucleotide (via a covalent bond), usually within CpG dinucleotides and in CpG-rich regions, called CpG islands, located within gene promoters or enhancers. This reaction is catalyzed by enzymes called DNA methyltransferases (DNMTs). Lower DNA methylation levels (hypomethylation) are usually associated with increased gene expression, while higher levels of DNA methylation (hypermethylation) are linked with decreased gene expression or even gene silencing [1].

Histone modifications include post-translational acetylation, phosphorylation, ubiquitination and methylation. Histones are acetylated at the lysine residues, via the action of histone acetyltransferases (HATs), and deacetylated by histone deacetylases (HDACs). Higher levels of histone acetylation can lead to the “opening of chromatin” (less tight wrapping of the DNA around nucleosomes), thus increasing the accessibility of DNA to the transcriptional machinery, while lower levels of histone acetylation lead to more tight and compact chromatin structures, as well as lower gene expression. Histones are methylated at lysine or arginine residues, a process that is catalyzed by histone methyltransferases. Chromatin condensation and the subsequent gene expression levels due to histone methylation vary depending on the location of the Lys/Arg residue and on the number of methyl groups being added [1].

Small non-coding RNAs are RNA sequences lower than 200 nucleotides in length, they include microRNAs (miRNAs), piwi-interacting RNAs (piRNAs), small interfering RNAs (siRNAs) and small nucleolar RNAs (snoRNAs), and they regulate transcriptional and translational processes.

Allergic diseases are a condition in which the body’s immune system reacts abnormally to typically harmless substances. These substances, called allergens, can cause an inflammatory immune response in some individuals. Common allergens include pollen, dust mites, pet dander, certain foods, insect venom and various medications [2]. Allergic reactions are defined as type I hypersensitivity reactions and are usually mediated by immunoglobulin E (IgE). These are preceded by a process called allergic sensitization that occurs upon first exposure to an allergen and encompasses a complex interaction of immune cells (epithelial cells and antigen-presenting cells—predominantly dendritic cells, T-helper cells and B cells) and allergens during epithelial/mucosal exposure, leading to an overproduction of allergen-specific immunoglobulin E (IgE) [3]. Upon repeated exposure, allergen-specific IgE binds to its receptors on mast cells and other effector cells, causing the release of histamine and other chemicals, which triggers the immediate- and late-phase hypersensitivity reactions via the activation and recruitment of effector cells, as well as additional production of IgE [4].

Allergic diseases, such as asthma, rhinitis, atopic dermatitis and food allergies, are among the most common illnesses and their prevalence rates are on the rise, leading to substantial healthcare costs. Adequate diagnostics and management of allergic diseases are crucial because in many cases, the course of the disease progresses (especially if not treated) and, additionally, allergic diseases may significantly affect the patient’s quality of life [5]. Allergen avoidance, antihistamines or corticosteroids, and allergen-specific immunotherapy are commonly used to treat allergies and desensitize the immune system to specific allergens [6,7].

In recent years, epigenetics has become a special focus in research involving the pathophysiology of allergic diseases. The epigenetic code provides plasticity of gene expression in response to environmental changes and allows for more rapid phenotypic adaptations across generations and during the lifetime of an individual, as certain epigenetic modifications (DNA methylation and histone modifications) are known to be passed down through multiple generations. The finding that familial allergy risk is sex-specific (i.e., allergic mothers are more likely to have allergic daughters and allergic fathers are more likely to have allergic sons) suggests an essential role of epigenetic risk factors for the disease [8,9,10]. Prenatal and early life influences likely play a significant role in the development and clinical manifestations of allergic diseases, as has been shown in other conditions [11]. The well-established “window of opportunity” in the development of the immune system includes maternal and early life environmental exposure, such as maternal infections, maternal and early life microbiota (gut and respiratory), and dietary and pollutant exposures that drive epigenetic changes and are essential for immune modulation and regulation. DNA methylation and histone modifications that modify immune responses (such as the shift toward Th2 cell response and decrease in Treg differentiation) are known to be involved in both risk of and protection from allergic diseases. Moreover, evidence shows that these epigenetic modifications change in association with the microbial agent involved (viral and bacterial, e.g., pathogen or probiotic and parasites, such as helminths) and probably play a role in the pathophysiology of specific asthma or allergy phenotypes [12].

The aim of this paper is to provide a comprehensive review of environmental and other factors influencing epigenetic modifications that are associated with the development and specific clinical features of allergic diseases.

This paper encompasses a review of the following exposure effects on epigenetic modifications in allergy:

- The effects of maternal and in utero exposure
- The effects of exposure to tobacco smoke
- The effects of diet
- The effects of microbial exposure
- The effects of exposure to air pollution
- The effects of exposure to persistent organic pollutants, such as pesticides
- The effects of other (intrinsic) in utero exposure
- The effects of early life events, such as viral infections in infancy
- Differential expression of small non-coding RNAs in allergy
- Epigenetic changes in treatment outcomes in allergic diseases (primarily asthma).

## 2. Environmental Exposure and Epigenetic Regulation in Allergic Diseases

The increase in the prevalence of allergic diseases seems to parallel the vast changes in our environment (e.g., pollution, climate changes, etc.). A number of large-scale epigenome-wide association studies (EWAS) have been undertaken and highlight the role of epigenetic regulation in mediating the effects of the changing environment on cellular homeostasis and immune modulation, which contribute to the development of asthma and allergic diseases [13]. Changes in DNA methylation have been associated with atopy and total serum IgE levels [14]. Additionally, differential DNA methylation has been reported in individuals with asthma, atopic dermatitis, food allergy and seasonal allergic rhinitis [14,15,16,17,18] when compared to healthy controls. Moreover, in the case of seasonal allergic rhinitis, changes in DNA methylation seem to vary during as well as outside of the allergy season (e.g., pollination season for inhaled pollen allergens) [18]. An EWAS study on cord blood identified 9 CGIs (CpG islands, key methylation sites) and 35 differentially methylated regions associated with the development of asthma later in life. Although different studies report different results, certain loci have been consistently associated with allergic traits, including the gene encoding SMAD3, an integral part of the transforming growth factor-beta (TGF-b) signaling pathway and an important regulator of T-cell differentiation [19]. Given all of this and the fact that certain epigenetic modifications are known to be stable and even transmitted over several generations, other than being a consequence of disease pathophysiology, it is likely that these changes precede the onset of allergic conditions and are one of the disease-causing mechanisms.

### 2.1. The Effect of Maternal and In Utero Exposure on Epigenetic Modifications

Maternal exposures, such as cigarette smoke, have already been shown to alter fetal lung development and immune function, contributing to an increased risk of respiratory disease [20,21,22]. Significant intrauterine exposures, including maternal diet and microbial exposure, are known to alter the risk of allergic disease in offspring. Studies show that prenatal exposures (diet, microbial infections, tobacco smoke and other pollutants) can epigenetically activate or silence immune-related genes, with significant effects on immune programming [23,24,25,26]. DNA methylation and histone modifications may control immune programming related to pro-allergic IL-4, IL-13 or counter-regulatory IFN-γ production [27,28,29,30]. Recent findings have also further described the induction of FOXP3, a Treg-specific transcription factor, and its dependence on DNA methylation and histone modifications in susceptibility to allergic disease, particularly at the maternal–fetal interface [31]. Strong evidence shows that early immune development is altered in children with allergic diseases. These alterations result in dysregulated immunity characterized by inadequate interferon-gamma production, modified innate immunity and a low network of regulatory T cells (Treg), culminating in a propensity for an uncontrolled T helper (Th) 2 immune response. These differences are already evident at birth, suggesting that prenatal factors may be crucial in the alternative programming of neonatal immunity [32]. These mechanisms are known to control both Th1 and Th2 cell differentiation and are also a prerequisite for FOXP3 expression and Treg differentiation [33]. During pregnancy, complex immune mechanisms develop that allow the fetal and maternal immune systems to co-exist. The maternal cellular immune system subtly adjusts to a “Th2 state” during pregnancy to reduce the immune responses to fetal antigens mediated by Th1 IFN-γ cells. This is achieved by suppressing fetal IFN-γ production through hypermethylation (gene silencing) of the IFN-γ gene promoter in CD4 1 T cells at the maternal–fetal interface. FoxP3 cells are also attracted to the maternal–fetal interface by human chorionic gonadotropin (HCG). Evidence shows that the FoxP3 gene is expressed at lower levels in the placenta of allergic women (and allergic children) [10]. Neonatal immunity reflects these maternal events, with reduced Th1 function and relative dominance of Th2 activity, with underlying epigenetic changes controlling these gene expression patterns. This has led to speculation that factors that increase gene methylation may increase disease risk by silencing signaling pathways (Th1 and Treg differentiation) that usually inhibit allergic Th2 differentiation and propensity to allergic respiratory disease [34].

**Figure 1:**
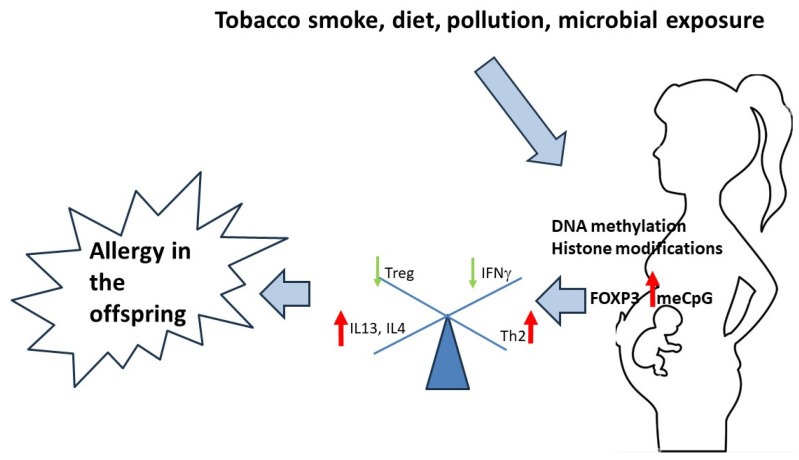
A summary of the effects of environmental exposure on epigenetic changes at the maternal–fetal interface that can lead to specific allergic pathophysiological mechanisms.

Infections in the mother, the use of antibiotics, the consumption of raw or unprocessed cow’s milk and contact with molds and pets have also been associated with an increased risk of allergic respiratory diseases. Certain aspects of maternal diet, maternal stress and exposure to pollutants, improved hygiene, inadequate exposure to microorganisms and the modern Westernized diet can affect cellular homeostasis, and these factors can affect epigenetic regulation [35,36]. A schematic representation of the effects of environmental exposure on epigenetic changes and the pathophysiology of allergic diseases is shown in Figure 1.

### 2.2. Exposure to Tobacco Smoke

In the prenatal period, maternal environmental influences can significantly affect lung development. There is strong evidence that maternal smoking during pregnancy as well as prolonged exposure to tobacco smoke in early infancy has adverse effects on lung development. These include increased airway responsiveness, smooth muscle layer thickness and collagen deposition. Altered DNA methylation patterns were observed in several genes in buccal cells from children exposed to tobacco smoke in utero, which may be a likely mechanism for increased disease risk [14]. Exposure to tobacco smoke in utero is associated with changes in DNA methylation at gene-specific (e.g., the *CYP1A1*, *AHRR* and *IL10* loci) and global levels and with an increased risk for childhood asthma and other allergic conditions, which also extends to e-cigarette smoke [37,38]. Additionally, evidence shows that even exposure to second-hand smoking in pregnancy increases the risk for the development of asthma in the offspring [39]. Moreover, epigenetic changes caused by tobacco smoking seem to have a transgenerational effect, as fathers who started smoking before or during puberty have an elevated risk for asthma in the offspring, likely due to epigenetic changes in precursor sperm cells [40].

### 2.3. Epigenetic Modifications and the Effects of Diet

One of the first epigenetic models in allergic diseases focused on the role of folic acid in the pathogenesis of these conditions, based on the ability of folic acid to epigenetically alter gene expression through its role as a methyl donor for DNA [13]. Supplementation of pregnant mice with a diet rich in folate resulted in altered gene methylation patterns and decreased transcriptional activity in the lung tissue of the offspring, with increased airway hyperresponsiveness, airway eosinophilia and production of chemokine and macrophage inflammatory proteins [41]. One of the genes involved was runt-related transcription factor 3, which has a protective role in the development of respiratory diseases via the induction of FOXP3+ Treg cells. Conversely, several studies reported that folic acid supplementation during pregnancy was associated with an increased risk of asthma in infants. However, the results of these studies should be considered in the context of other related nutrients, such as vitamins B2, B6, B12, methionine and choline [42,43]. The consumption of polyunsaturated fatty acids (PUFA) may influence epigenetic changes and contribute to protection against allergen sensitization or the development of allergic diseases [44]. Maternal vitamin D supplementation induces changes in DNA methylation in infants, specifically genes involved in collagen metabolism and apoptosis. Moreover, in monocytes derived from patients with asthma, pretreatment with vitamin D increases glucocorticoid receptor binding to the dual-specificity phosphatase 1 (*DUSP1*) promoter and increased histone acetylation at the GRE (glucocorticoid response elements), resulting in decreased airway inflammation [45].

### 2.4. Effects of Microbial Exposure to Epigenetic Regulation in Allergic Diseases

Recently, the interaction between the external and internal (for example, gut microbiome) environments and epigenetic regulation has become a special focus in studying the pathophysiologic mechanisms of allergic diseases. A number of studies demonstrated the relationship between changes in the biodiversity of gut, skin and airway microbiota with allergic diseases [46,47,48]. Moreover, the microbiome influences epigenetic regulation, cell differentiation and polarization, as well as homeostasis [49]. Short-chain fatty acids produced by commensal microorganisms may serve as signaling molecules in the processes of DNA methylation and histone modifications [50]. Although early postnatal exposure (mostly in the first six weeks of life) is considered the most significant source of direct microbial exposure to the developing infant, it is becoming increasingly clear that the epigenetic influences of bacteria are already substantial in utero. In humans, exposure to a higher degree of microbial load in rural environments has been proven to have a protective role against the development of allergic diseases [51]. Studies have also shown that protection from the development of allergic diseases mediated by microbial exposure in utero is associated with increased Treg function in neonates, FOXP3 expression and decreased methylation of the FOXP3 gene [26]. Based on these observations, it has been proposed that microbial exposures may cause epigenetic changes that affect key immune gene expression patterns during critical periods of early life and development, thus contributing to predisposition for allergic diseases [52]. Additionally, microbiota may trigger Th17 and Treg immune responses and, moreover, their interaction with epithelial cells promotes the polarization of Th17 cells, essential for host immunity [53,54,55].

### 2.5. Exposure to Air Pollution and Epigenetic Effects in Allergic Diseases

Oxidative stress resulting from exposure to tobacco smoke and air pollution can have significant epigenetic effects by altering nuclear factor kB (NF-kB) activation or histone modification and chromatin remodeling of proinflammatory genes. As a potent source of oxidative stress, tobacco smoke (cigarette smoke) contributes to decreased histone deacetylase (HDAC) activity, leading to differential activation of NF-kB and expression of the proinflammatory cytokines IL-6 and IL-8 in peripheral lung tissue [12]. NF-kB is ubiquitously expressed in various cell types and can induce histone modifications that activate or silence inflammatory genes and other signal transduction pathways. During pregnancy, the induction of inflammatory genes can affect placental function and fetal programming. Oxidative stress resulting from chronic and high levels of exposure to traffic exhaust may also cause epigenetic changes during pregnancy. Studies in mice showed that exposure to diesel exhaust particles increased IgE production following sensitization to allergens (*Aspergillus fumigatus*) through hypermethylation of IFN-γ and hypomethylation of the IL4 locus [27]. Studies have reported that high maternal exposure to traffic exhaust is correlated with methylation of the long-chain acetyl-coenzyme A synthetase family member three and the development of allergic disease symptoms in children [56]. Elevated levels of air pollutants, such as polycyclic aromatic hydrocarbons (PAHs), affect epigenetic modifications, such as increased DNA methylation at the FOXP3 locus in peripheral blood mononuclear cells, which is associated with elevated total serum IgE [57]. Air pollution has been shown to alter the expression profile of miRNAs involved in inflammation associated with the development of allergic diseases [58,59].

### 2.6. Exposure to Persistent Organic Pollutants and Epigenetic Effects

Organic products from industry and agriculture (including polychlorinated biphenyl compounds, organochlorine pesticides, dioxins and phthalates) contaminate modern homes, food, clothing and water sources and accumulate in human tissues with age. Although they have immunosuppressive effects in humans at high doses, low concentrations of these pollutants may more selectively inhibit Th1 immune responses and promote allergic Th2 immune responses through their “estrogenic” hormonal activity [60]. Some of these products have already been detected in breast milk, umbilical cord blood and placental tissue, indicating that they may affect early development. Studies have shown that persistent organic pollutants (especially organochlorine pesticides) are present in 94% of fat samples from mothers who have undergone caesarean section and in 62% of breast milk samples, which may be associated with immune reactions in both the mother and the newborn, especially the allergic inflammatory response [11,61]. Pesticides and other pollutants have also been linked to epigenetic effects, including differential methylation specific for each active ingredient (pesticide-specific methylation signals) and positive epigenetic age acceleration [62,63,64].

### 2.7. Other Intrauterine Factors That Affect Epigenetic Modifications and Allergic Disease Risk

In the case of allergic diseases, the maternal phenotype is a known risk factor for diseases in the child. Maternal allergic status has a much stronger influence on allergic diseases and Th1 IFN-γ production in the newborn than paternal allergies [65]. Studies have also shown that maternal allergy status alters immune interactions between the mother and the fetus and reduces Th1 activation to the human leukocyte antigen mismatch of fetal alloantigens [66]. Many other changes in the intrauterine environment can directly affect gene expression in the placenta and potentially alter the offspring’s phenotype. Pre-eclampsia, the use of corticosteroids (and other medications, such as antibiotics) and stress have been associated with epigenetic changes affecting gene expression, placental immune function, growth retardation and congenital disabilities [67]. Maternal stress is critical for gene expression in the placenta, as adrenal glucocorticoid production modulates inflammatory gene expression via the hypothalamic–pituitary–adrenal axis, with recognized effects on glutathione metabolism and DNA methylation [68]. Inflammatory diseases during pregnancy can also alter the immune function of the placenta through the sex-specific production of cortisol [69]. The early induction of innate inflammatory genes in newborns (including IL-1b and TNFα) is closely linked to the later development of allergic diseases. These inflammatory mediators, important for immune programming, induce histone modifications and may be responsible for the alternative programming of neonatal immunity toward a pro-allergic response [70]. Additionally, maternal disease state, such as diabetes (gestational diabetes mellitus), has been associated with changes in DNA methylation, as well as immune dysregulation, increased inflammation and changes in the microbiome in the offspring [71]. A study involving a large number of patients showed that gestational diabetes is associated with an increased risk for allergic diseases in the offspring, including allergic rhinitis, urticaria and atopic dermatitis [72].

### 2.8. Early Life Events and Epigenetic Changes Affecting Allergic Diseases

After birth, environmental influences also affect local events in the developing airways. Lower respiratory tract infections associated with wheezing in the first year of life are a crucial risk factor for asthma development at the age of six, both in non-atopic and atopic children [73,74]. This suggests that viral-induced inflammation of the lower airways early in the postnatal development can have profound long-term effects that are more pronounced than events later in life. Studies have shown that RSV infection in infancy leads to alterations in histone methylation, resulting in decreased proinflammatory cytokine production and an increase in Th2 cytokine production. Additionally, RSV infection alters DNA methylation of certain genes within the TGF-b superfamily, resulting in increased polarization of T cells toward a Th2 and Th17 phenotype. Moreover, decreased methylation of the perforin-1 enhancer (important in the control of viral infections) was found in children aged 3–4 years who had been hospitalized due to severe cases of RSV-induced bronchiolitis, which indicates that RSV may have a role in altering the immune response years after infection [75]. Adenoviral infections increase the expression of inflammatory genes via surface envelope proteins that may interact with histone modifiers and play a role in the pathogenesis of COPD. In general, viral pathogen infections have been associated with DNA methylation events in the host [76,77,78,79]. Early life viral infections can induce epigenetic changes of genes within inflammatory pathways that synergize with allergen sensitization and contribute to developing a Th2-high asthmatic phenotype [80].

### 2.9. Effects of Exposure to Heavy Metals on Epigenetic Changes in Allergy

Heavy metals, such as mercury, cadmium, arsenic, chromium, nickel, copper and lead, are well-established environmental pollutants, due to their longevity in the ecosystem, toxicity and low doses required to exhibit a toxic effect and, most importantly, due to their bioaccumulation in organisms over time. Exposure to heavy metals, especially in utero and during childhood, is known to be harmful for human health, having both direct toxic and cumulative hazardous effects in the development of chronic conditions. Exposure to heavy metals from water, air or food has been linked to neurological and cardiovascular diseases, cancer, diabetes, hepatotoxicity, nephrotoxicity and immune disorders. It also causes substantial epigenetic changes, and these effects are persistent, even lifelong. Exposure to chromium, mercury, nickel, arsenic and lead is known to cause both gene-specific and global DNA methylation changes [81,82,83], and it affects all types of histone modifications (acetylation, phosphorylation, ubiquitination and methylation). Exposure to arsenic has been known to cause shifts in the immune responses toward a proinflammatory state [84]. Exposure to lead affects B cell production, MHC activity and T-cell production and function [85,86], while chromium exposure has been linked to both types of allergic hypersensitivity (anaphylactic and delayed) as well as with allergic contact dermatitis, which occurs, among other mechanisms, via changes in the epigenome [87].

### 2.10. The Changing Environment and Epigenetic Changes in Allergic Diseases

In the past decades, the rapid and growing climate changes have paralleled the several-fold increase in the prevalence of allergic diseases. Climate changes include global warming due to greenhouse gases coming from various human activities (such as industry, traffic and deforestation), pollution (air, water and soil), and increases in the frequency of extreme weather events (floods, storms, wild fires, heat waves, droughts, snowstorms and blizzards, etc.), which lead to reduced biodiversity and health hazards, affecting both the prevalence of infectious diseases due to their effects on the food and water supply and the prevalence of non-communicable diseases, with allergies being one of the most prominent. For example, the rise in the global temperature and atmospheric CO_2_ favors the growth and spreading of the highly invasive plant species into areas they were never present before (such as ragweed), thus contributing to the rise in the prevalence of respiratory allergic diseases [88]. Specific atmospheric conditions and the increase in air pollution, as a consequence of climate change (such as higher ozone and NO_2_ levels, as well as attachment to particulate matter or micro- and nano-plastic particles), also enhance the allergenicity of certain airborne allergens [89]. Higher temperatures (heatwaves) have been associated with changes in DNA methylation and epigenetic aging (such as the Horvath clock), and allergic sensitization and asthma [84,90]. Exposure to heavy drought in utero has been linked with changes in DNA methylation (both hyper- and hypo-methylation of specific key genes), including the *IFNG* gene, a vital gene in the immune response and asthma pathogenesis [91]. Higher levels of precipitation and exposure to thunderstorms increase the level of exposure to certain allergens (such as molds) that are known triggers of asthma. Exposure to air pollution form wildfires (mostly fine particulate matter) is associated with changes in DNA methylation of 47 genes that are involved in immune regulation and inflammation, including the *HLA-DQB1* gene associated with asthma and the *LRRC43* gene associated with eczema and allergy [85]. Additionally, exposure to air pollution deriving from wildfires is associated with hypermethylation of *FOXP3* in children and impaired function of Treg cells, which are crucial for immune tolerance to allergens [92].

### 2.11. Expression of Small Non-Coding RNAs and Allergic Diseases

**Figure 2:**
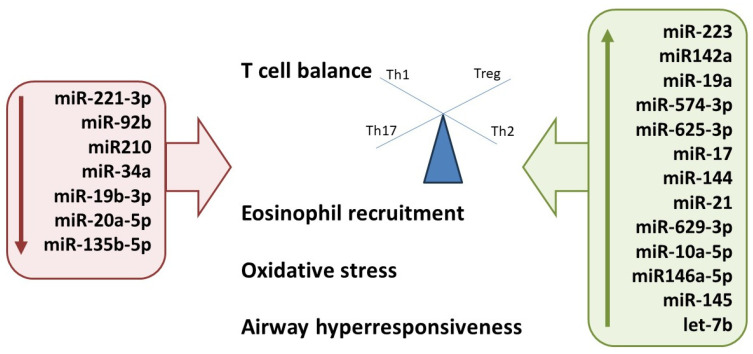
A schematic representation of differential expression of miRNAs involved in the pathogenesis of allergic diseases.

In patients with allergic diseases, miRNAs play a crucial role in the development, differentiation, maturation and activation of immune cells, and remodeling and deregulation of the airways [93]. There is evidence that miRNA expression differs in tissues, cells and biological fluids of allergic and healthy patients [94,95,96,97]. Since it is unclear whether the expression of non-coding RNA (ncRNA) molecules (miRNA, siRNA and long non-coding RNA) is transmissible across generations, it is more likely they serve as a more direct regulatory mechanism for gene expression patterns. For example, studies have indicated that let-7 miRNAs (a highly conserved group of at least nine miRNAs known to be involved in Toll-like receptor 4 (TLR4) signaling) may play a proinflammatory role, as targeted inhibition of let-7 miRNA suppressed Th2 cytokine production, lung eosinophil recruitment and airway hyperreactivity in in vivo models [98]. In addition, significantly increased levels of miR-145, miR-21 and let-7b have been reported in allergic airways, especially in allergic airways triggered by house dust mites. Studies have also shown that miR-146 miRNAs (miR-146a and miR-146b) and miR-223 are upregulated in asthma and other allergic diseases [99,100]. A complex network of miRNAs, such as miR-155, miR-21, miR-29 and miR-375, rather than individual miRNA expression changes, are involved in the regulation of the Th1/Th2 balance and could, therefore, play a significant role in the development of allergic diseases [101]. Air pollution alters both circular and local airway miRNA expression involved in important immunomodulatory mechanisms in asthma, such as autophagy, oxidative stress, NF-κB signaling, apoptosis and epithelial to mesenchymal transition [102]. In utero exposure to tobacco smoke was also associated with changes in the expression of miR-223 in maternal and cord blood, as well as with decreased Tregs and asthma development [103]. Exposure to inhaled particulate matter has also been associated with increased levels of expression of miR-155 in the serum of children with asthma [104]. Additionally, differential miRNA expression has been found in mammalian cell lines when exposed to heavy metals [87]. A schematic representation of differential miRNA expression patterns is shown in Figure 2.

**Figure 3:**
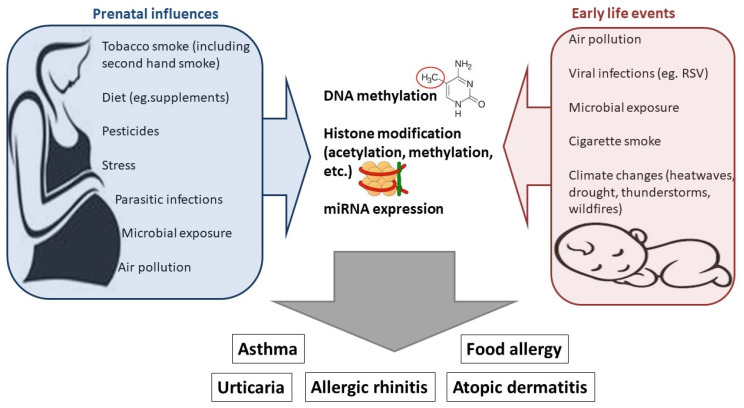
A summary of prenatal and early life influences on the risk for the development of allergic diseases mediated by epigenetic modifications.

A schematic representation of the epigenetic mechanisms involved in mediating maternal and early postnatal exposures is shown in Figure 3.

## 3. Epigenetic Changes and Treatment Outcomes in Allergic Diseases

Different allergic diseases have different clinical manifestations within the same epithelial barrier organ (phenotypes), driven by distinct pathophysiological and molecular endotypes. Phenotyping of inflammatory profiles aims at enabling personalized treatment of allergic diseases. Identifying epigenetic changes associated with these phenotypes and endotypes can contribute to better management and possible prevention of allergic diseases, to the assessment of tolerance after immunotherapy and to the prediction of treatment success in early phases [1,105,106,107].

It is also evident that both stable and transient epigenetic modifications (DNA methylation, histone modifications and ncRNA expression) mediate environmental exposure in the development of asthma and the clinical features of the disease [11]. Therefore, epigenetic regulation has emerged as a potential mechanism for the effect of asthma-related pharmacological therapies. For example, inhaled corticosteroids (ICS), which have been used for decades to treat inflammation in acute and chronic asthma and chronic obstructive pulmonary disease (COPD), are thought to act, at least in part, via certain epigenetic modifications, such as histone acetylation and, according to recent studies, miRNA expression [108,109]. Corticosteroids bind intracellularly to glucocorticoid receptors, which are activated and bind to glucocorticoid response elements in the promoter regions of glucocorticoid-responsive genes. Corticosteroids exert their anti-inflammatory effects in part by inducing histone acetylation of anti-inflammatory genes (e.g., mitogen-activated protein kinase phosphatase-1 (MKP-1)) and by recruiting histone deacetylases (HDAC2) and causing deacetylation of proinflammatory genes (e.g., IL-8, NF-κB and activator protein-1 (AP-1)). In addition, low concentrations of theophylline, another drug commonly used for asthma treatment (usually administered to treat exacerbations), have been shown to reverse the effects of corticosteroid resistance by restoring HDAC2 activity, possibly via selective inhibition of phosphoinositide 3-kinase (PI3K)-δ and phosphorylation of downstream kinases [110,111].

In addition, another proposed mechanism of corticosteroid action could act via the induction of miRNA expression [112], through increased or decreased expression of miRNAs on a tissue-specific level. Although the expression of specific genes (including miRNAs) varies widely across tissues, and although a given asthma treatment acts via more localized mechanisms (ICS), the treatment can still elicit a systemic response (to a relative extent), which is reflected in the bloodstream. Indeed, blood transcriptomics has been used successfully, even in diseases confined to the brain [112]. A CAMP study revealed that several miRNA expression profiles (hsa-miR-155-5p and hsa-miR-532-5p) are associated with long-term treatment outcomes in children with asthma [109].

## 4. Environmental Exposure, Epigenetic Changes and Protection from Allergy

As discussed in Section 2.4., microbial exposure, both early in the postnatal development or in utero, seems to greatly affect the offspring’s susceptibility to allergic diseases. Other than microbial exposure, infections with other protozoa and parasites, such as helminths, seem to have an important role in immune modulation and development. This is reinforced by the fact that a great part of the immune responses involved in the defense from parasitic infections and allergic diseases overlap. Certain pattern recognition pattern members participate in the response to parasitic (e.g., helminth) infection and are responsible for driving T-cell polarization. Adult helminths secrete certain antigens that are known to activate a number of immune effector cells, including basophils, mast cells, eosinophils, Th2 cells and innate lymphocyte T cells 2 (ILC2), as well as induce the production of innate and adaptive cytokines. Additionally, lipids derived from helminths can stimulate the production of a number of inflammatory cytokines, such as TNFα, IL-6, IL-8, IL-10 and IL-12. Moreover, in vivo studies in mice demonstrated that maternal exposure to helminths seems to carry a protective effect against the development of allergic diseases, which is predominantly mediated by maternal IFN-γ [12,112].

Other than differential DNA methylation, other epigenetic modifications have been associated with protection against the development of allergic diseases or the development of immune tolerance, such as decreased histone acetylation of Th2 genes in a murine food allergy study assessing acquired immune tolerance associated with raw milk consumption. Additionally, early life consumption of raw milk and breastfeeding seems to elicit a protective effect against the development of allergy via changes in the expression of miRNAs (extracellular or exosomal) involved in Treg and Th2 cell regulation and activation [113].

## 5. Conclusions

A number of environmental factors have been associated with the risk for allergic diseases, which are mediated by global and gene-specific epigenetic variations in multiple tissues. Epigenetic modification plays a crucial role in immune regulation and the pathophysiology of different clinical manifestations of allergic diseases. Although the specific epigenetic mechanisms involved in the development and clinical features of allergic diseases are not fully understood, a number of attempts have been made to shed better light on the complex interaction of environmental exposures and allergy, as well as specific disease phenotypes, including large-scale epigenome-wide association studies (EWAS), such as the Pregnancy and Childhood Epigenetics (PACE) and the Mechanisms of the Development of Allergy (MEDALL). A number of studies have shown that specific disease-associated phenotypic traits may be inherited transgenerationally, and are accompanied by changes in DNA methylation patterns, providing a possible explanation for the transgenerational effects of environmental exposure and the changing environment, as well as for the several-fold increase in the prevalence rates of allergic diseases in the last three to four decades. Other epigenetic modifications, such as histone modifications and differential miRNA expression, have been repeatedly associated with specific allergy endotypes (such as the T2-high asthma), while fewer have been linked to protective factors against the development of allergic diseases, such as microbial exposure, parasitic infections, breastfeeding, and maternal supplementation with specific nutrients during pregnancy and breastfeeding. Although a clear causal role has not yet been established, it is more than likely that epigenetic mechanisms are not merely a consequence of the pathophysiology of allergy, but rather have a predisposing role in the development of allergic diseases. Future studies are needed to provide better insight into the complex epigenetic regulation in allergic diseases and, in the near future, complement or replace current diagnostic procedures. Recent studies also indicate that epigenetic mechanisms might play a role in the prediction of treatment outcomes, which may ultimately lead to the optimization of current treatment regimes, especially in cases of poor response to medication. It is also reasonable to believe that these findings will also drive the development of new therapeutic modalities and novel prevention strategies aimed at the epigenetic control of the shift in effector cell subpopulations characteristic of allergic diseases.

## Data Availability

Not applicable.
